# A Rare Case of Granulomatous Mastitis Associated With Positive Systemic Lupus Erythematosus Serology in a Young Female Patient: A Case Report and Literature Review

**DOI:** 10.7759/cureus.33279

**Published:** 2023-01-02

**Authors:** Mohammed S Abdalla, Eltaib Saad, Ahmed Abdulrahman, Ahmed A Abdulrahman, Mousab Mokhtar, Khalid Mohamed

**Affiliations:** 1 Internal Medicine, Ascension Saint Francis Hospital, Evanston, USA

**Keywords:** granulomatous mastitis, lobular granulomatous inflammation, rare disease, autoimmune diseases, systemic lupus erythematosus

## Abstract

Granulomatous mastitis (GM) is a rare benign breast disease that affects women of childbearing age, usually within five years of pregnancy. The hallmark diagnostic feature of GM is the presence of lobular granulomatous inflammation. The occurrence of this clinicopathological entity is usually idiopathic. Nevertheless, GM has often been associated with systemic inflammatory conditions of either infectious (such as tuberculosis) or autoimmune etiology (particularly sarcoidosis, vasculitis, and less likely systemic lupus erythematosus [SLE]).

In this report, the authors described an unusual case of GM that was associated with features of SLE in a young female patient who presented with a painful breast lump. Histopathological examination of the lump’s biopsy showed GM. Further laboratory workup revealed evidence of some immunological criteria of SLE. Steroid therapy led to the resolution of the patient’s breast swelling. The breast mass remained in remission with hydroxychloroquine treatment. Only a handful of similar cases in the current literature demonstrated a plausible association between SLE and GM. Our case provides a reference to consider SLE as a possible differential diagnosis when GM is encountered in young-aged female patients.

## Introduction

Granulomatous mastitis (GM) was first described as a separate clinicopathological entity by Kessler and Wolloch in 1972 [1] when they described five cases of mastitis that were characterized by the presence of lobular granulomas and abscess formation. The clinical presentation of those five patients masqueraded as neoplastic breast tumors. The occurrence of this clinicopathological entity is usually idiopathic. Nevertheless, GM has often been associated with systemic inflammatory conditions of either infectious (such as tuberculosis) or autoimmune etiology (particularly sarcoidosis, vasculitis, and less likely systemic lupus erythematosus [SLE]) [2-5].

Herein, the authors described an unusual case of a young female who presented with a breast lump that was initially suspicious of a neoplastic process and was eventually diagnosed with GM in association with some features concerning SLE. The pathophysiology and the clinical presentation of GM were provided in the communication with a brief review of cases of GM associated with either SLE or SLE serology that had been published in the current literature.

## Case presentation

A 32-year-old female patient with no significant past medical history presented for evaluation of a right breast lump that she started noticing two weeks prior to her presentation. The patient did not report any history of breast trauma. The lump was painless and not associated with nipple discharge. The patient has not reported any history of joint pains or swelling, fevers, weight loss, loss of appetite, skin rashes, hair loss, or oral or genital ulcers. The patient denied prior or current use of hormonal contraceptives. She has never used tobacco, alcohol, or illicit drugs. The patient does not have any family history of breast cancer or other breast diseases and has no family history of autoimmune conditions. Regarding gynecological history, the patient had three term pregnancies; the last pregnancy was six years before her presentation and is currently not lactating. She has intrauterine device for contraception for the last year and has used oral contraceptive pills for five years before that.

Physical examination revealed erythematous skin changes overlying the right breast and an irregular 5 cm x 4 cm tender mass over the right upper quadrant of the right breast; no nipple inversion or skin retraction was noted. There was no palpable axillary or cervical lymph node enlargement. Examination of the left breast and axilla showed no masses or skin changes. The remainder of the physical examination was unremarkable.

Initial laboratory evaluation, including complete blood count (CBC), revealed mild anemia (Hb of 11.7; 12.0-15.5 g/dL), mild leukopenia (white blood cell [WBC] of 3.1 (4.2-11.0 K/mcL), and platelets count of 179 (140-450 K/MCL), The complete metabolic panel (CMP) including kidney function was within normal limits.

A triple assessment was completed for the breast lump with imaging and histopathological examination. Mammography showed an indeterminate equal density of focal asymmetry in the right breast. Breast ultrasound revealed a 3.3 cm x 2.6 cm irregular fluid collection with an irregular internal wall in the right breast central to the nipple in the retro-areolar area; color flow images demonstrated cutaneous hyperemia.

**Figure 1:**
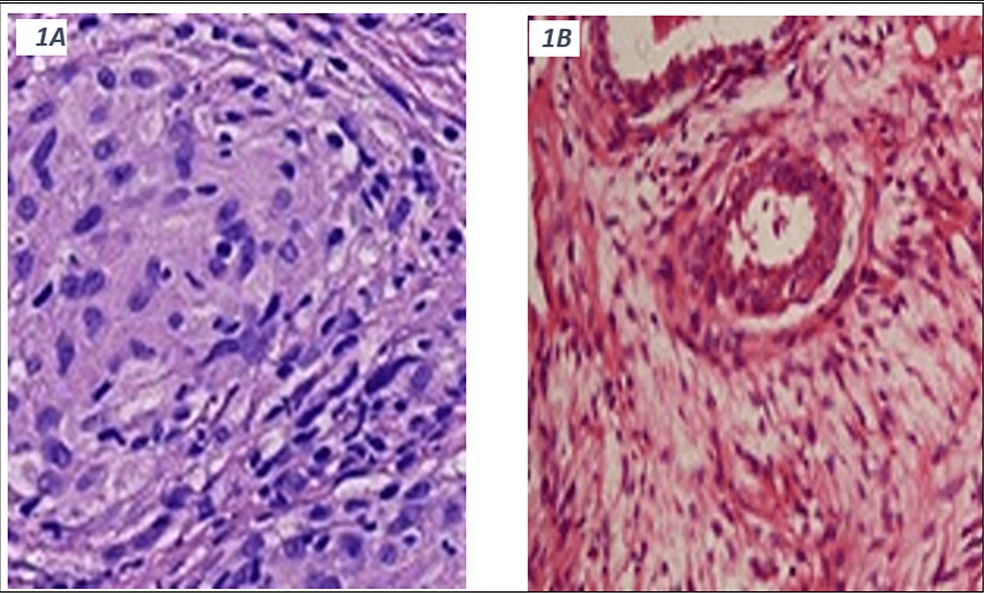
(A) Hematoxylin and eosin (H&E) stained histopathological image (x100) and (B) immunohistochemically stained section of the breast biopsy showing granulomatous inflammation

Attempted imaging-guided aspiration was unsuccessful, and the patient was treated empirically with a 10-day course of antibiotics for presumed bacterial mastitis; however, there was no clinical improvement after the completion of treatment. Given the persistent mass, the patient underwent a core biopsy. Histopathology demonstrated breast tissue with acute and chronic inflammation and suppurative granulomatous inflammation consistent with GM (Figure 1, Panels A and B). Bacterial cultures and special stains for acid-fast bacilli (AFB) and fungi were negative. Given the presence of granulomatous inflammation and the lack of an underlying infectious etiology of mastitis, an autoimmune disease was suspected. Therefore, further laboratory workup was pursued. The autoimmune panel revealed a positive speckled antinuclear antibody (ANA), and the reflex testing demonstrated a positive anti-double-stranded DNA antibody (anti-ds DNA): 11 (<9 units/mL) and low complement levels (C3: 69 [79-152 mg/dL] and C4:11 [16.0-38.0 mg/dL]). Other autoimmune antibody screening was negative (myeloperoxidase antibody, IgG < 0.2 [<1.0 AI] and serine protease 3 antibody, IgG < 0.2 [<1.0 AI]).

The patient was started on high-dose prednisone and hydroxychloroquine as induction therapy for a suspected SLE. The patient’s breast lump showed a remarkable response to the medical therapy. Azathioprine was added for maintenance therapy along with hydroxychloroquine as steroids were eventually tapered off, and the breast mass completely disappeared on follow-up breast ultrasound within a few weeks of starting immune suppressive treatment. In addition, her CBC and complement levels were normalized. The patient regularly follows up with ophthalmology and rheumatology with no report of further recurrence of symptoms while being on hydroxychloroquine and azathioprine and report of symptoms of medication-related adverse effects.

## Discussion

GM is a rare benign inflammatory condition of the breast of unknown etiology [1-8]. Although some risk factors have been reported, including tobacco smoking, hormonal imbalance, and alpha-1 antitrypsin deficiency, the vast majority of cases are idiopathic, hence the name idiopathic granulomatous mastitis (IGM). The condition commonly presents with a hard breast mass which in many cases is associated with inflammation of the overlying skin and sometimes sinus tract formation [9].

GM must be differentiated from other forms of granulomatous breast inflammation, including the granulomatous variants of duct ectasia and plasma cell mastitis, where the lobular spread is inconspicuous [4,6,9]. It should also be differentiated from the known causes of granulomatous inflammation, such as sarcoidosis and infections associated with granuloma formation. Fungal and mycobacterial stains should be performed in all cases of GM [9]. The microbiological cultures were negative in the case of our patient, and the infectious workup was unrevealing.

Although no clear etiopathogenetic factors have been identified for GM, several studies have suggested immunological disturbances. In Ozel et al.’s case series [9], six out of eight GM patients tested positive for rheumatoid factor (RF), and two of the six tested positive for ANA and anti-ds DNA antibodies [9] (Table 1). Although none of these patients had other clinical manifestations suggestive of autoimmune diseases, there have been rare case reports linking GM with full-blown SLE [10] (Table 1). GM has also reportedly presented within weeks to a few months of diagnosis of other autoimmune disorders, such as erythema nodosum and inflammatory arthritis [11,12], which further supports an immunologic etiology for the condition.

**Table 1:** Published cases with a possible association between GM and SLE or immunological markers of SLE GM: Granulomatous mastitis; SLE: Systemic lupus erythematosus; ANA: Antinuclear antibody.

Author, Year	Age/Gender	Status of Prior SLE Diagnosis	Clinical Course	Treatment Response
Sellitto et al., 2013 [13]	65/Female	No prior diagnosis of SLE	Presentation with multifocal breast lumps masquerading clinically and radiologically as breast cancer. Laboratory workup revealed positive serology for anti-ANA, anti-dsDNA, and anti-phospholipid antibodies as well as prolonged aPTT. No other clinical criteria for SLE.	The breast lesions were responsive to corticosteroids therapy.
Zhang et al., 2014 [10]	22/Female	Known case of SLE on corticosteroids and hydroxychloroquine with a stable disease status	Presentation with hyperprolactinemia and recurrent flare-ups of GM upon self-discontinuation of immunosuppressive therapy for SLE.	Remission of recurrent GM and hyperprolactinemia with re-institution of corticosteroids and hydroxychloroquine therapy and treatment with bromocriptine.
Ozel et al., 2012 [9]	Two out of eight female patients with GM had positive anti-ANA and anti-dsDNA antibodies, and both patients’ breast lumps responded well to the corticosteroid therapy. No further clinical details were provided regarding the presence of other clinical features of SLE at the time of GM diagnosis, but none of them were previously diagnosed with SLE.			

SLE is rarely associated with mastitis as part of lupus panniculitis, which often involves the breasts; however, this typical lupus mastitis is rare, and only a few cases have been reported in the literature [14-20]. In addition to the well-characterized cases of lupus, ANAs were detected in 50 out of 78 patients with non-lactational mastitis in one study [21].

GM must be distinguished from lupus mastitis by the absence of the typical histological features of lupus mastitis, including hyaline fat necrosis and linear deposition of IgG and C3 along vessel basement membranes and the dermo-epidermal junction [15], and by the presence of a lobular granulomatous inflammatory response rather than lobular panniculitis on histopathological examination [2,22].

This presented patient has not met the diagnostic criteria of SLE according to the American College of Rheumatology (ACR) criteria [7]. Nevertheless, the presence of bi-cytopenia (mild anemia and leukopenia) along with the positivity for ANA and dsDNA as well as low complement levels were highly suggestive of an immunological process associated with her GM, particularly SLE. This possible immune etiology was further supported by the complete resolution of her breast lump and the normalization of the blood cell counts and complement levels after starting immunosuppressive therapy. Furthermore, the lack of recurrence of the breast lump while on maintenance immunosuppressive therapy also strongly favors the plausible association between GM and the presumed diagnosis of SLE in our patient as reported in a previous similar case by Zhang et al. [10]. In the latter-referenced patient, the GM recurred when maintenance SLE immunosuppressive therapy was self-discontinued, and the GM resolved with the re-institution of corticosteroids and hydroxychloroquine [10].

There is no consensus yet regarding the standard management of GM, owing to the possible diverse systemic inflammatory conditions that are often associated with GM [9,13]. Further diagnostic workup to differentiate the heterogenous associated conditions (including autoimmune conditions and infectious etiologies) would help guide the appropriate treatment of GM. Our case, in line with the previous similar published cases in the available literature, suggests that an autoimmune screening, including ANA and dsDNA, is justified to look for a possible underlying autoimmune etiology.

Furthermore, the exclusion of granulomatous infections such as tuberculosis and endemic fungal infections is vital prior to the initiation of immune suppressive therapy as demonstrated in our presented case. Our case also supports the cautious use of immune suppressive therapy in suspected and confirmed cases of SLE as part of the medical management of GM [15], as surgical treatment is associated with potential complications such as fistula formation, cosmetic problems, and increased risk of recurrence [23-26].

## Conclusions

In this article, the authors described a case of GM in a young female patient who presented with a painful breast lump. The diagnosis of GM in our patient was associated with positive immunological markers of SLE, suggesting a plausible association between GM and autoimmune connective tissue diseases, such as SLE, as has been reported in other cases. Induction immune suppressive therapy led to a remarkable resolution of the breast mass, which remained in remission on maintenance therapy, consistent with the similar patients reported in the reviewed literature. Despite the rare association of GM with autoimmune diseases, a further diagnostic workup for possible underlying systemic autoimmune conditions, as demonstrated in our case and other similar cases described in this communication, would be appropriate to help the management of GM.

## References

[1] Kessler E, Wolloch Y (1972). Granulomatous mastitis: a lesion clinically simulating carcinoma. Am J Clin Pathol.

[2] Going JJ, Anderson TJ, Wilkinson S, Chetty U (1987). Granulomatous lobular mastitis. J Clin Pathol.

[3] Al-Khaffaf B, Knox F, Bundred NJ (2008). Idiopathic granulomatous mastitis: a 25-year experience. J Am Coll Surg.

[4] Maffini F, Baldini F, Bassi F, Luini A, Viale G (2009). Systemic therapy as a first choice treatment for idiopathic granulomatous mastitis. J Cutan Pathol.

[5] Tuli R, O'Hara BJ, Hines J, Rosenberg AL (2007). Idiopathic granulomatous mastitis masquerading as carcinoma of the breast: a case report and review of the literature. Int Semin Surg Oncol.

[6] Néel A, Hello M, Cottereau A (2013). Long-term outcome in idiopathic granulomatous mastitis: a western multicentre study. QJM.

[7] Barreto DS, Sedgwick EL, Nagi CS, Benveniste AP (2018). Granulomatous mastitis: etiology, imaging, pathology, treatment, and clinical findings. Breast Cancer Res Treat.

[8] Larsen LJH, Peyvandi B, Klipfel N, Grant E, Iyengar G (2009). Granulomatous lobular mastitis: imaging, diagnosis, and treatment. AJR Am J Roentgenol.

[9] Ozel L, Unal A, Unal E (2012). Granulomatous mastitis: is it an autoimmune disease? Diagnostic and therapeutic dilemmas. Surg Today.

[10] Zhang LN, Shi TY, Yang YJ, Zhang FC (2014). An SLE patient with prolactinoma and recurrent granulomatous mastitis successfully treated with hydroxychloroquine and bromocriptine. Lupus.

[11] Salesi M, Karimifar M, Salimi F, Mahzouni P (2011). A case of granulomatous mastitis with erythema nodosum and arthritis. Rheumatol Int.

[12] Olfatbakhsh A, Beheshtian T, Djavid GE (2008). Granulomatous mastitis, erythema nodosum, and oligoarthritis in a pregnant woman. Breast J.

[13] Sellitto A, Santoriello A, De Fanis U (2013). Granulomatous lobular mastitis: another manifestation of systemic lupus erythematosus?. Breast J.

[14] Ashton MA, Lefkowitz M, Tavassoli FA (1994). Epithelioid stromal cells in lymphocytic mastitis--a source of confusion with invasive carcinoma. Mod Pathol.

[15] Kinonen C, Gattuso P, Reddy VB (2010). Lupus mastitis: an uncommon complication of systemic or discoid lupus. Am J Surg Pathol.

[16] Warne RR, Taylor D, Segal A, Irish A (2011). Lupus mastitis: a mimicker of breast carcinoma. BMJ Case Rep.

[17] Lucivero G, Romano C, Ferraraccio F (2011). Lupus mastitis in systemic lupus erythematosus: a rare condition requiring a minimally invasive diagnostic approach. Int J Immunopathol Pharmacol.

[18] Sanders LM, Lacz NL, Blackwood MM, Ongcapin E, Santos-Zabala ML (2012). Lupus mastitis without systemic disease: unusual imaging findings including MRI. Breast J.

[19] Mosier AD, Boldt B, Keylock J, Smith DV, Graham J (2013). Serial MR findings and comprehensive review of bilateral lupus mastitis with an additional case report. J Radiol Case Rep.

[20] Tanaka Y, Manabe H, Shinzaki W, Hashimoto Y, Komoike Y (2020). A case of lupus mastitis in a patient with systemic lupus erythematosus. Breast J.

[21] Xu R, Guo QQ, Yang LP, Lai ML, Tong L (2016). [Variations of peripheral blood autoantibody, immunoglobulin, and complement levels in patients with non-lactational mastitis and their clinical significances]. Nan Fang Yi Ke Da Xue Xue Bao.

[22] Kaviani A, Zand S, Karbakhsh M, Ardalan FA (2017). Synchronous idiopathic granulomatosis mastitis and breast cancer: a case report and review of the literature. Arch Breast Cancer.

[23] Mazlan L, Suhaimi SN, Jasmin SJ, Latar NH, Adzman S, Muhammad R (2012). Breast carcinoma occurring from chronic granulomatous mastitis. Malays J Med Sci.

[24] Mahlab-Guri K, Asher I, Allweis T, Diment J, Sthoeger ZM, Mavor E (2015). Granulomatous lobular mastitis. Isr Med Assoc J.

[25] Asoglu O, Ozmen V, Karanlik H (2005). Feasibility of surgical management in patients with granulomatous mastitis. Breast J.

[26] Sakurai K, Fujisaki S, Enomoto K, Amano S, Sugitani M (2011). Evaluation of follow-up strategies for corticosteroid therapy of idiopathic granulomatous mastitis. Surg Today.

